# Quarantine Hospital. N. Y.

**Published:** 1847-08

**Authors:** 


*Quarantine Hospital. N. Y.*—From the Annalist of July 1, we learn that the number of patients received at the Quarantine Hospital, N. Y. from May 31st, to June 1st, was 524; 402 sick of Typhus, of whom 13 died. From June 7th to 11th, there remained, of Typhus, cases, 584; deaths 11; in hospital, all told, 741; new admissions 289. From June 11th to 21st, there remained in hospital, of Typhus, cases, 616, death 16; new admissions 208; total number in hospital 641. Dr. Morley, jr. and Dr. Langer have been ill, but are recovering.

